# P-1681. Determining Appropriateness of Pneumonia Treatment in Long-Term Care Facilities Pilot Project

**DOI:** 10.1093/ofid/ofae631.1847

**Published:** 2025-01-29

**Authors:** Cullen Adre, Dipen Patel, Callyn Wren, Quynh Nhu Dao, Christopher Wilson, Christopher D Evans

**Affiliations:** Tennessee Department of Health, Nashville, Tennessee; HAI/AR, Tennessee Department of Health, Nashville, Tennessee; Tennessee Department of Health, Nashville, Tennessee; AmPharm, Parsons, Tennessee; Tennessee Department of Health, Nashville, Tennessee; Tennessee Department of Health, Nashville, Tennessee

## Abstract

**Background:**

In 2019 IDSA updated the guidelines for community-acquired pneumonia (CAP). To date there are limited AU data in long-term care facilities (LTCFs). The Antimicrobial Appropriateness for Pneumonia in LTCFs (AAPL) Project was developed to determine which antibiotics are most used and their appropriateness in treating pneumonia (PNA) in TN LTCFs using a modified National Antimicrobial Prescribing Survey (NAPS) tool.

**Methods:**

We recruited five LTCFs to collect retrospective PNA data from January 2023 to February 2023. Pharmacy students collected data for residents meeting a proposed PNA surveillance definition or with a written PNA diagnosis and entered into a REDCap database. Cases were reviewed and scored by two independent reviewers with an appropriateness score based on the NAPS tool (5 = not assessable, 3/4 = inappropriate, and 1/2 = appropriate). Descriptive statistics were used to analyze AAPL data and analyses performed on SAS Version 9.4.

**Results:**

Thirteen cases were identified and reviewed; two were excluded due to no antibiotics received in the LTCF. Every chart had a written PNA diagnosis with 54.6% CAP and 18.2% HAP. 17.7% of cases had positive chest X-ray, 15.7% had acute decline in at least one daily living activity, and 11.8% had leukocytosis. 84.6% of cases (n=10) had MRSA risk factors). The most common antibiotics used were amoxicillin/clavulanate, ceftriaxone, and levofloxacin. The average duration of therapy was 7 days. 54.5% of antibiotics were appropriate (n=6), 36.4% were inappropriate (n=4), and 9% were not assessable (n=1). Nine cases had agreement on appropriateness, while two cases needed a third adjudicator to review.

**Conclusion:**

The most common antibiotics used reflect current PNA guidelines, however only 54.5% were labeled as appropriate. Average duration was found to be longer than 2019 guideline recommendations (5 days of treatment with clinical stability). The data and experience from the pilot can be used to focus stewardship efforts on shorter durations with clinical stability, improve the survey tool, and build facility specific reports to improve prescribing practices with current PNA guidelines.

**Disclosures:**

**All Authors**: No reported disclosures

